# Immunomagnetic Sex-Sorting of Landrace Boar Semen Using a Porcine Y-Specific scFv Antibody: Efficiency and Post-Sorting Sperm Quality

**DOI:** 10.3390/ani16070998

**Published:** 2026-03-24

**Authors:** Apinya Satsook, Marninphan Thongkham, Surat Hongsibsong, Anucha Sathanawongs, Phanuwit Paitoon, Chaiwat Arjin, Pornchai Rachtanapun, Korawan Sringarm

**Affiliations:** 1 Office of Research Administration, Chiang Mai University, Chiang Mai 50200, Thailand; apinya.satsook@cmu.ac.th; 2 Department of Animal and Aquatic Sciences, Faculty of Agriculture, Chiang Mai University, Chiang Mai 50200, Thailand; marninphan.t@cmu.ac.th (M.T.); phanuwit_pai@cmu.ac.th (P.P.); chaiwat.arjin@cmu.ac.th (C.A.); 3 School of Health Sciences Research, Research Institute for Health Sciences, Chiang Mai University, Chiang Mai 50200, Thailand; surat.hongsibsong@cmu.ac.th; 4 Department of Veterinary Biosciences and Veterinary Public Health, Faculty of Veterinary Medicine, Chiang Mai University, Chiang Mai 50100, Thailand; anucha.sa@cmu.ac.th; 5 Faculty of Agro-Industry, Chiang Mai University, Chiang Mai 50200, Thailand; pornchai.r@cmu.ac.th

**Keywords:** porcine sexing semen, scFv antibody, semen quality

## Abstract

This study evaluated the efficiency of an scFv antibody-based sex-sorting method using HL magnetic microbeads for the separation of X- and Y-chromosome-bearing sperm in Landrace boar semen, as well as its effects on sperm quality parameters. A dose-dependent plateau in H4L4 antibody coupling efficiency was observed, with 2 mg identified as the optimal concentration, consistently achieving approximately 50% sperm-binding efficiency across boars. Sex ratio analysis confirmed effective discrimination between X- and Y-bearing sperm, with the X-enriched fraction showing a high proportion of X-sperm (77–81%) and the Y-enriched fraction exhibiting high Y-sperm purity (>83%). CASA revealed a significant effect of the sex-sorting process on sperm kinematics (*p* < 0.001). X-enriched sperm maintained physiological motility, with total and progressive motility comparable to CON, whereas the Y-enriched fraction showed a marked decline in kinematic performance. Flow cytometric analysis demonstrated that membrane integrity remained high in CON and X-enriched semen (>81%), while a significant reduction in viability was observed in the Y-enriched fraction (50.85%; *p* < 0.001). Consistently, mitochondrial membrane potential analysis indicated pronounced physiological stress in Y-enriched sperm, with significantly reduced mitochondrial activity compared to CON and X-enriched fractions. No significant differences were detected among individual boars (*p* > 0.05). In conclusion, this study demonstrates that HL magnetic bead-based sperm sexing effectively separates X- and Y-sperm in chilled Landrace boar semen, while preserving the quality of X-enriched sperm.

## Simple Summary

In pig production, being able to control the sex of piglets can improve farm efficiency and animal welfare. This study evaluates an antibody-based method to separate X- and Y-chromosome-bearing sperm in Landrace boar semen using magnetic microbeads. These methods successfully enriched both fractions, resulting in a high proportion of analysis of X-enriched sperm in the supernatant fraction (unbound fraction) and Y-enriched fraction in the elute fraction. Sperm quality was then assessed after sorting using computer-assisted sperm analysis and imaging flow cytometry. The X-enriched sperm maintained good motility and sperm integrity, comparable to control semen. In contrast, the Y-enriched fraction showed reduced motility, lower viability, and decreased mitochondrial function, indicating that the sorting and elution steps may impose physiological stress on Y-sperm. Overall, these findings confirm that the magnetic microbead antibody approach could discriminate between X- and Y-sperm in boar, but further optimization is required to preserve the quality of sperm in the Y-enriched fraction before the technique can be applied widely in the swine industry.

## 1. Introduction

The global swine industry, valued at billions of dollars annually, serves as a cornerstone of agricultural economies and global food security [1,2]. However, the sector faces formidable challenges, most notably catastrophic losses from disease outbreaks such as African Swine Fever (ASF). As a highly contagious and lethal pathogen, ASF has decimated porcine populations worldwide, causing profound disruptions to production chains and economic stability [3]. Consequently, rebuilding sow herds has become a critical priority for mitigating economic impact and ensuring the long-term sustainability of swine operations. The demand for female piglets has surged, as they are indispensable for expanding breeding herds and restoring population growth following disease-induced losses [4,5].

Beyond productivity, the industry is increasingly pressured by ethical concerns regarding the surgical castration of male piglets. Traditionally performed to enhance meat quality and curb aggressive behavior, this practice has faced mounting criticism for failing to meet modern animal welfare standards [6]. Therefore, the development of sex-selection technologies presents a dual opportunity to optimize production efficiency and address these pressing ethical considerations. While sex-sorted semen is a well-established technology in the cattle industry, enabling high-precision production of female calves for dairy or males for beef [7], its application in swine has been hampered by technical and economic barriers. Fluorescence-activated cell sorting (FACS), which differentiates X-chromosome-bearing spermatozoa (X-sperm) and Y-chromosome-bearing spermatozoa (Y-sperm) based on DNA content, offers high accuracy and is widely utilized in bovine breeding [8,9]. However, in the swine industry, FACS remains largely impractical due to high operational costs, the inherent complexity of the sorting process, and diminished sperm viability resulting from mechanical and chemical stressors [10]. Furthermore, the high sperm dosage required for porcine insemination limits the scalability of FACS, as the process cannot yet produce sufficient quantities of viable cells efficiently. Although advancements in flow cytometry have achieved 80–90% accuracy in sex prediction [11], the resulting spermatozoa often exhibit reduced fertility, rendering the method commercially unviable for large-scale pig farming.

Thongkham et al. (2025) [12] developed an scFv antibody generated against Y-sperm antigens derived from Duroc boar semen. Previous evaluations demonstrated that this scFv antibody exhibited 100% cross-reactivity with Y-sperm and only 4.14% cross-reactivity with X-sperm, indicating strong specificity and consistent binding capability to porcine spermatozoa. Furthermore, when applied in combination with PLA microbeads, the H4L4 scFv antibody efficiently separated Y-sperm from X-sperm in porcine semen. This approach yielded a Y-enriched fraction containing 78.4% Y-sperm and an X-enriched fraction with up to 76.01% X-sperm, while maintaining sperm quality for up to three days of storage [13]. These findings highlight the potential of the H4L4 scFv-based system as a promising and innovative method for porcine sperm sexing.

However, antigenic expression on the sperm plasma membrane may vary among pig breeds, which could influence antibody binding efficiency and separation performance. Therefore, it remains necessary to determine whether an scFv antibody developed from Duroc sperm can be effectively applied to other commercially important breeds. In particular, Landrace boars are extensively used in breeding programs, yet data regarding the binding specificity and cross-reactivity of this scFv antibody toward Landrace X- and Y-sperm are limited. Accordingly, the objective of this study was to investigate the efficiency of separating X- and Y-chromosome-bearing sperm from Landrace pigs, using scFv antibodies bound to microbeads, and to evaluate the impact of this method on sperm quality parameters.

## 2. Materials and Methods

### 2.1. Chemicals and Reagents

The single-chain variable fragment (scFv) antibody targeting male-specific antigens located on the plasma membrane of Y-chromosome-bearing spermatozoa (H4L4 scFv) was generated and prepared in our laboratory. This antibody exhibited strong binding affinity toward Y-sperm, while demonstrating minimal cross-reactivity with X-sperm (4.25%). Magnetic poly(lactic acid) particles with carboxyl-functionalized surfaces (PLA-M; micromod Partikeltechnologie GmbH, Rostock, Germany) were used in this study. The particles were spherical, with an average diameter of approximately 30 µm (range: 20–50 µm), a solid content of 10 mg/mL, and a density of 1.3 g/cm^3^. N-hydroxysuccinimide (NHS), 1-ethyl-3-(3-dimethylaminopropyl) carbodiimide hydrochloride (EDC), Dulbecco’s phosphate-buffered saline (DPBS), and 3,3′,5,5′-tetramethylbenzidine (TMB) were obtained from Sigma-Aldrich (St. Louis, MO, USA). SYBR-14, propidium iodide (PI), Hoechst 33342, and peanut agglutinin lectin from Arachis hypogaea conjugated with Alexa Fluor 488 (PNA-Alexa 488) were purchased from Invitrogen (Eugene, OR, USA). Anti-HA tag peroxidase antibody was supplied by Abcam (San Diego, CA, USA).

### 2.2. Semen Sample Collection

All procedures involving experimental animals were reviewed and approved by the Animal Care and Use Committee of Agricultural Animal Science, Faculty of Agriculture, Chiang Mai University (approval no. AG05001/2569). Ejaculates were collected from three Landrace boars aged between two and three years with proven fertility and routinely used in artificial insemination programs and processed individually at the Faculty of Animal Science and Technology, Maejo University, Chiang Mai, Thailand. Only semen samples meeting strict quality criteria—specifically, a sperm concentration exceeding 200 × 10^6^ cells/mL and total motility above 80%—were selected for subsequent experiments. The sample size was determined based on previous studies in porcine sperm sex-sorting and practical limitations related to animal availability. Individual boars were considered as biological replicates, while repeated ejaculates collected from each boar were treated as within-animal replicates.

### 2.3. Optimizing the Coupling of H4L4 Antibodies to Magnetic Microbeads

The experimental procedures were developed based on the method previously described by Thongkham et al. (2025) [13], with minor modifications. PLA-M magnetic microbeads (1 mg) were chemically activated using 0.1 mM EDC in combination with 0.1 mM NHS. The activated beads were then incubated with H4L4 scFv antibody solutions at final concentrations of 0, 2, 4, and 8 mg/mL, generating four experimental preparations. All reactions were carried out at 4 °C with gentle agitation and allowed to proceed overnight. Following incubation, the beads were isolated using a neodymium magnetic separator, after which the supernatant was removed. Residual reactive carboxyl groups on the bead surface were quenched by treatment with Tris buffer. The beads were subsequently washed with DPBS and resuspended in 100 µL of DPBS for storage at 4 °C until further use in semen sexing assays. The efficiency of antibody coupling was indirectly evaluated based on the proportion of sperm bound to the beads after incubation.

Fresh porcine semen was diluted with M III^®^ extender to a final concentration of 10 × 10^6^ spermatozoa/mL. Each HL beads preparation was added to the diluted semen and incubated for 20 min at 37 °C under gentle mixing. Magnetic separation was then performed by placing the samples on a neodymium magnet for 5 min at room temperature, allowing the collection of bead-bound sperm. The supernatant containing unbound spermatozoa (supernatant fraction) was carefully removed.

To recover spermatozoa attached to HL beads, the bead–sperm complexes were treated with an elution buffer consisting of Tris–citric acid extender supplemented with 0.05 mM imidazole (pH 7.4) and incubated for 20 min at 37 °C. The elution buffer (Tris–citric acid supplemented with imidazole) was used to disrupt antibody–antigen interactions, facilitating the release of Y-bearing sperm from the bead surface. The samples were subsequently exposed to a magnetic field to immobilize the microbeads, and the released sperm fraction (eluted fraction) was transferred to fresh tubes. Both bound and unbound fractions were retained for evaluation of HL beads binding efficiency. Binding efficiency (%) was calculated as the proportion of sperm recovered in the eluted fraction relative to the total sperm count prior to separation.

### 2.4. Production of Sexed Sorted Porcine Semen by HL Beads

Fresh semen samples from three Landrace pigs were collected five times per boar and were divided into the following experimental groups:

- For the control treatment (CON), a semen sample containing 500 × 10^6^ cells was incubated with 50 mg of PLA-M magnetic microbeads (not conjugated to scFv antibodies) at room temperature (RT) for 15 min; then, sperm were collected into new tubes. Unconjugated magnetic beads were used as a control to assess non-specific binding.
- For sperm sexing with HL beads, 500 × 10^6^ cells of semen were incubated with 50 mg of PLA-M magnetic microbeads conjugated with 0.2 mM EDC/NHS and 2 mg/mL scFv antibody at RT for 15 min. The scFv beads, along with the captured Y-sperm, were separated using a strong neodymium magnet. Unbound sperm were collected into new tubes as the supernatant fraction, while Y-sperm entrapped on the scFv-beads were released by incubation in elution buffer for 15 min at RT and transferred to new tubes as the eluted fraction.

**Figure 1:**
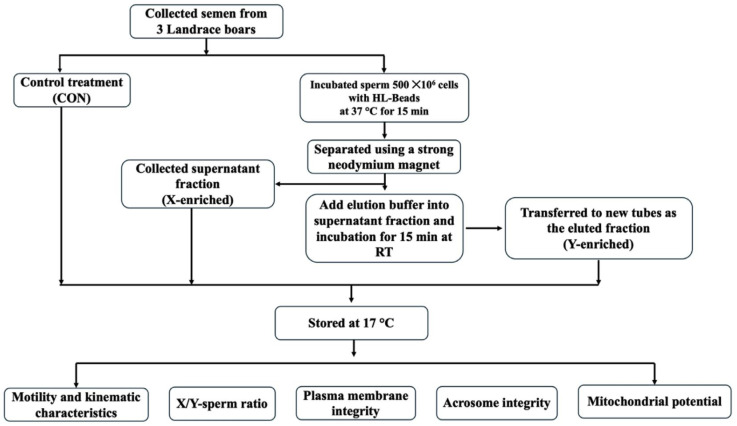
Schematic illustration of the HL beads procedure used for fresh swine semen sex-sorting.

Based on the separation process, two sperm fractions were obtained: (i) the X-enriched fraction, represented by the unbound sperm collected in the supernatant fraction after HL bead incubation; and (ii) the Y-enriched fraction, obtained from sperm released from the scFv-conjugated HL beads following elution (elute fraction). All treated sperm samples were stored at 17 °C for sperm quality testing. The overall workflow of the HL beads-based swine semen sex-sorting procedure is summarized schematically in Figure 1.

### 2.5. Discrimination of X/Y-Sperm Ratio

The proportion of X- and Y-chromosome-bearing spermatozoa was assessed in accordance with the method previously reported by Thongkham et al. (2021) [14]. Briefly, semen samples were adjusted to a final concentration of 2 × 10^6^ sperm/mL using phosphate-buffered saline (PBS) and labeled with Hoechst 33342 at a final volume of 1.2 µL (50 µg/mL). Staining was performed at 37 °C for 10 min under light-protected conditions. Following staining, the samples were subjected to analysis using an imaging flow cytometer (FlowSight). Excitation of the Hoechst fluorochrome was achieved with a 405-nm laser operating at 15 mW. Fluorescence intensity distributions were subsequently generated, enabling discrimination and quantification of X- and Y-sperm populations.

### 2.6. Evaluation of Plasma Membrane Integrity

Sperm viability in semen straws obtained from each experimental group was evaluated using a dual-fluorescence staining approach. Live spermatozoa were identified with SYBR-14, whereas membrane-compromised cells were detected using propidium iodide (PI). Semen samples were adjusted to a final concentration of 2 × 10^6^ sperm/mL in phosphate-buffered saline (PBS) and incubated for 10 min at room temperature under light-protected conditions with 1.2 µL of diluted SYBR-14 and 3 µL of PI (2.4 mM). Following staining, membrane integrity was determined by quantifying the proportions of SYBR-14-positive and PI-positive spermatozoa. Analyses were conducted using an imaging flow cytometer (FlowSight, Seattle, WA, USA), with excitation of both fluorochromes achieved using a 488-nm laser operating at 60 mW. Data collection and subsequent analysis were performed with IDEAS software (version 6.2; Amnis, Seattle, WA, USA) [15].

### 2.7. Mitochondrial Membrane Potential

Aliquots from each experimental group were labeled with the lipophilic cationic dye 5,5′,5,5′-tetrachloro-1,1′,3,3′-tetraethylbenzimidazolylcarbocyanine iodide (JC-1; Molecular Probes) at a final concentration of 12 µM (from a 3-mM stock solution). Staining was carried out for 30 min at 37 °C under light-protected conditions. After incubation, mitochondrial membrane potential was evaluated using an imaging flow cytometer (FlowSight). Data collection and subsequent analysis were conducted with IDEAS software (version 6.2; Amnis, Seattle, WA, USA) [16].

### 2.8. Evaluation of Sperm Motility and Kinematic Variables Using Computer-Aided Sperm Analysis (CASA)

Sperm motility and kinematic properties of semen from the control (CON), X-enriched, and Y-enriched fractions were evaluated at 37 °C using a computer-assisted sperm analysis system (CASA). Motion characteristics were assessed with AndroVision software (Minitube of America—MOFA^®^, Verona, WI, USA). For analysis, 4.7 µL aliquots of pre-warmed semen were introduced into a Hamilton 2X-CEL^®^ chamber slide (Hamilton Thorne Inc., Beverly, MA, USA) and examined on a slide warmer maintained at 37 °C. For each sample, at least 10 microscopic fields were recorded, corresponding to a minimum of 500 spermatozoa. The CASA system quantified both motility and kinematic parameters, including total motility (TM, %), progressive motility (PM, %), average path distance (DAP, µm), curved line distance (DCL, µm), straight line distance (DSL, µm), average path velocity (VAP, µm/s), curved line velocity (VCL, µm/s), straight line velocity (VSL, µm/s), and beat cross frequency (BCF, Hz) [17].

### 2.9. Statistical Analysis

Data obtained for X/Y-sperm ratio, sperm viability, acrosomal status, and sperm motility and kinematic parameters were subjected to statistical evaluation using a randomized complete block design (RCBD). The analysis included three blocks, corresponding to individual boars (Boar 1, Boar 2, and Boar 3), and three experimental treatments (control, X-enriched, and Y-enriched groups). To assess the normality of the data, the Shapiro–Wilk normality test was performed as part of the univariate procedure. This test confirmed that the data followed a normal distribution, justifying the use of parametric tests for further analysis. Differences among treatments (control, X-enriched, and Y-enriched groups) were analyzed using one-way ANOVA followed by Tukey’s HSD test for multiple comparisons. All statistical analyses were performed with SPSS software (version 20.0; SPSS Inc., Chicago, IL, USA). Statistical significance was accepted at *p* < 0.05. Results are expressed as mean ± standard deviation (SD).

## 3. Results

### 3.1. Efficiency of H4L4 Antibody Coupling to Magnetic Microbeads

**Figure 2:**
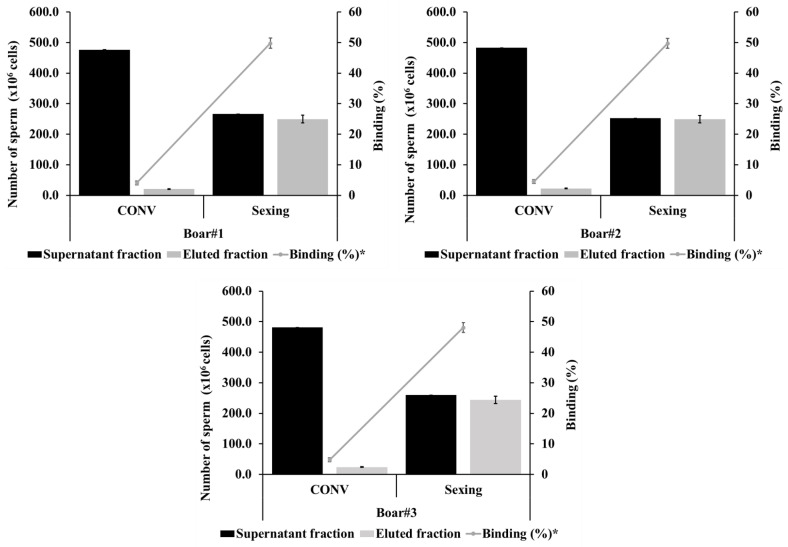
Comparative analysis of sperm fractions and antibody binding percentages between conventional (CON) and sexed-sorted spermatozoa across three different boars. * % Binding = (Eluted fractionsperm/total sperm) × 100.

The experimental results demonstrate a clear dose-dependent plateau in the efficiency of H4L4 antibody coupling to magnetic microbeads for sperm sexing applications (Table 1). Based on the quantitative analysis, the 2 mg/mL antibody concentration is identified as the optimal capacity, achieving a binding efficiency of 50.83%, which effectively bifurcates the total sperm population into balanced supernatant and eluted fractions. Notably, increasing the antibody concentration to 4 mg/mL and 8 mg/mL yielded marginal to negligible changes in binding percentages (50.73% and 48.89%, respectively), indicating that the magnetic microbeads reached steric saturation at the 2 mg/mL threshold. The tripartite graphical data across Boars 1, 2, and 3 further validates this observation, showing a highly consistent distribution pattern where the Sexing group consistently maintains a binding rate of approximately 50% compared to the CON (Figure 2) This high level of reproducibility across different biological replicates confirms that the 2 mg/mL concentration provides the most cost-effective and biologically stable configuration for high-purity sperm separation (Table 2).

### 3.2. Discrimination Between X- and Y-Sperm After Sexing by HL Magnetic Beads

The data shows that the sex-sorting process successfully shifted the sperm ratio compared to the natural distribution (CON), which remained near a 50:50 balance for all boars. In the supernatant fraction, there was a significant increase in X-sperm, reaching approximately 77% to 81%. Conversely, the eluted fraction became highly enriched with Y-sperm, particularly for Boars 1 and 2, where levels exceeded 83% (Table 3). These results indicate that the sorting method is effective at separating X and Y chromosomes into different fractions, allowing for a more controlled selection of the desired sex in chilled boar semen.

### 3.3. Sperm Motility in Sexed Porcine Semen

The quantitative assessment of Computer-Assisted Sperm Analysis (CASA) parameters demonstrates a highly significant discriminatory impact (*p* < 0.001) of the sexing process on the kinematic integrity of porcine spermatozoa, revealing a clear disparity between X- and Y-enriched populations (Table 4). The data indicate that X-enriched sperm successfully maintain physiological parameters, with total motility (TM: 76.67 ± 4.13%) and progressive motility (PM: 70.14 ± 2.62%) remaining statistically comparable to the CON. Conversely, the Y-enriched fraction exhibited a pronounced deleterious response to the separation process, evidenced by a substantial decline in curvilinear VCL and VSL, alongside a significant reduction in BCF and HAC. Importantly, the absence of statistically significant differences among the experimental blocks (Boars 1, 2, and 3; *p* > 0.05) demonstrates the robustness and reproducibility of the procedure. This finding indicates that the observed physiological changes are attributable to the sex-sorting treatment itself, rather than to biological variability among individual boars.

### 3.4. Sperm Membrane Integrity in Sexed Porcine Semen

**Figure 3:**
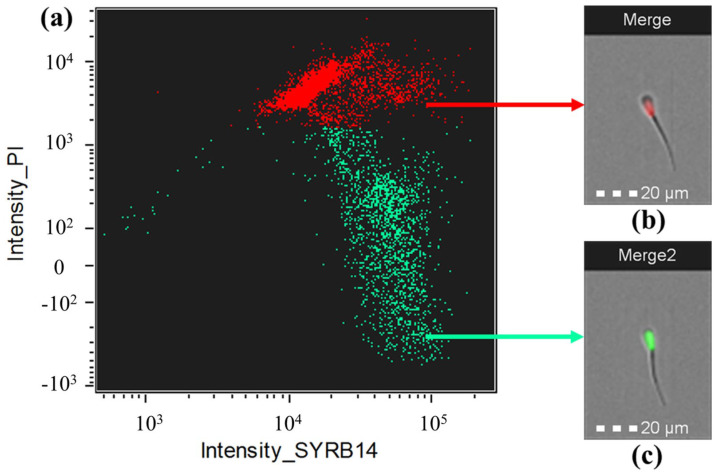
Sperm viability was assessed after the sexing process. (**a**) Flow cytometry dot plot showing sperm populations based on SYBR-14 (live sperm; green) and propidium iodide (PI; dead sperm; red) fluorescence intensity. The upper population represents non-viable (PI−positive) sperm, while the lower population represents viable (SYBR−14−positive) sperm. (**b**) Representative fluorescence image of non-viable sperm stained with PI (red). (**c**) Representative fluorescence image of viable sperm stained with SYBR−14 (green). Scale bar = 20 µm.

The flow cytometry plot supports these findings by clearly separating healthy, live sperm (shown in green) from damaged, dead sperm (shown in red) based on their membrane integrity (Figure 3). The experimental data reveal a significant impact of the sex-sorting process on the physiological quality of boar semen. According to Table 5, the CON and X-enriched groups maintained high membrane integrity (>81% living cells), while the Y-enriched treatment resulted in a substantial decline in viability, with living cells dropping to 50.85%. This sharp decrease is statistically significant (*p* < 0.001). Interestingly, the individual boar factor showed no significant difference (*p* > 0.05), indicating that the negative effect on sperm quality is consistent across different subjects and is primarily driven by the sorting treatment itself rather than individual biological variation.

### 3.5. Sperm Mitochondrial Membrane Potential in Sexed Porcine Semen

According to Table 6, the proportion of spermatozoa with high and low MMP was significantly affected by treatment (*p* < 0.001). CON and X-enriched semen did not differ significantly, as both showed similarly high proportions of MMP-high and low proportions of MMP-low sperm (in contrast, Y-enriched semen exhibited a marked reduction in MMP-high sperm and a substantial increase in MMP-low sperm. No significant block effect was observed among boars for either MMP-high or MMP-low sperm populations (*p* = 0.083 and *p* = 0.069, respectively), with relatively consistent MMP profiles across all individuals.

## 4. Discussion

Sperm sex-sorting technology is a valuable tool for improving productivity, economic efficiency, and sustainability in livestock production [18]. In porcine systems, the ability to control offspring sex supports targeted breeding strategies, particularly the efficient production of replacement females and improved herd management [19]. This approach not only enhances production efficiency but also addresses animal welfare concerns by reducing the generation of unwanted animals [20]. In addition, the use of sex-sorted semen from genetically superior pigs can accelerate genetic progress and improve feed efficiency, contributing to more sustainable swine production systems [21,22]. Immunological sorting represents a promising, non-invasive, and cost-effective alternative to flow cytometry [23]. This approach relies on antibodies targeting sperm membrane antigens to selectively affect specific sperm populations [24]. Compared with flow cytometry, immunological methods can yield higher sperm numbers per dose and are therefore more suitable for large-scale commercial application. Immunological sorting has gained increasing attention for its application in animal sperm sex-sorting [25]. In cattle, antibodies targeting sex-specific sperm antigens have been successfully applied in combination with magnetic-activated cell sorting (MACS), resulting in an effective and scalable sex-sorting approach [15]. Recently, immunological sexing approaches have been developed and successfully applied in pigs. However, these methods have primarily been evaluated using Duroc boars, which served as the reference breed for antibody production. Consequently, it remains necessary to assess the applicability and robustness of this immunological sex-sorting strategy in other boar breeds, as breed-specific differences may influence sorting efficiency and subsequent production performance. Importantly, the antibody used in this study (H4L4 scFv) was originally developed using sperm antigens derived from Duroc boars. As such, its binding specificity and efficiency may be influenced by potential breed-related variations in sperm membrane antigen expression. Differences in genetic background could affect antigen availability, antibody–sperm interaction, and, ultimately, the efficiency of immunological separation. Therefore, it is essential to evaluate whether this Duroc-derived antibody can be effectively applied to other breeds. In this context, the present study specifically addresses the cross-breed applicability of the immunological sex-sorting approach by assessing its performance in Landrace boars, thereby providing insight into the generalizability of the method beyond the original breed. In the present study, Y-scFv was successfully coupled to the surface of PLA-M magnetic microbeads, with concentrations of 2, 4, and 8mg/mL showing high-efficiency binding to Y-bearing sperm. At these concentrations, the proportions of sperm recovered in the supernatant and eluted fractions were comparable, indicating effective separation performance. Based on these results, the 2 mg/mL Y-scFv concentration was selected for further evaluation in individual Landrace boars. Following immunological sex-sorting, similar proportions between the supernatant and eluted fractions were consistently observed across Landrace boars. Subsequently, PLA-M microbeads coupled with 2 mg/mL Y-scFv were used for semen production and immunological sex-sorting, followed by an assessment of post-sorting semen quality.

Assessing sperm quality is essential for the success of artificial insemination in pigs, as key parameters such as motility, morphology, and viability are closely associated with fertility potential [26]. High-quality semen is directly linked to improved farrowing rates and increased litter size, thereby enhancing farm productivity and economic efficiency [27]. Among these parameters, sperm motility is a critical determinant of male fertility, as successful fertilization depends on the ability of sperm to migrate through the female reproductive tract and respond appropriately to complex physiological and physical regulatory factors. Optimal motility not only facilitates sperm transport but also serves as a key selection criterion for morphologically normal and functionally competent sperm capable of capacitation and fertilization [28,29]. Consequently, semen sex-sorting procedures require careful evaluation, as the sorting process can directly influence sperm motility, viability, and subsequent fertilization capacity [30,31]. The CASA analysis demonstrated a clear differential impact of the sex-sorting process on sperm kinematic parameters, with X- and Y-enriched fractions exhibiting distinct motility responses. Notably, X-enriched sperm maintained total and progressive motility at levels comparable to conventional semen. In contrast, the Y-enriched fraction showed a marked reduction in key velocity and flagellar-related parameters, including VCL, VSL, BCF, and HAC, indicating compromised flagellar activity and altered swimming patterns. These changes may reflect increased susceptibility of Y-bearing sperm to manipulation during immunological sex-sorting, potentially due to differences in metabolic capacity or sensitivity to antibody binding and magnetic separation forces. The flow cytometry analysis demonstrated that the sex-sorting process differentially affected the physiological integrity of boar sperm, with X-enriched semen largely maintaining membrane integrity and mitochondrial function comparable to conventional semen. In contrast, the Y-enriched fraction exhibited a pronounced reduction in both membrane integrity and mitochondrial membrane potential, indicating increased physiological stress associated with the sorting procedure [32]. The observed decline in Y-sperm quality during storage may be attributable to stresses imposed by the immunological sex-sorting process itself. During separation, magnetic microbeads bind to Y-sperm surface antigens, followed by an elution step to release sperm from the antibody–microbead complex, a sequence that may compromise membrane stability and cellular integrity [17,33]. Similar increases in sperm damage and cell death following immunological sex-sorting procedures have been previously reported by Thongkham et al. (2025) [13], supporting the notion that the sorting and elution steps can adversely affect sperm viability. Consequently, Y-enriched sperm may be more susceptible to quality deterioration during extended storage compared with X-enriched and conventional semen. Although both X- and Y-sperm enrichment were successfully achieved, a reduction was observed in motility, membrane integrity, and mitochondrial function in the Y-enriched fraction. This suggests that Y-bearing sperm may be more susceptible to stress during the sorting process, making the method currently more suitable for practical application using X-enriched semen. One possible explanation is that Y-bearing sperm differ in membrane composition or structural stability, rendering them more vulnerable to mechanical stress during bead binding and separation. Additionally, differences in metabolic activity or mitochondrial sensitivity may contribute to reduced resilience under experimental conditions, particularly during incubation and elution steps. Therefore, further optimization of the immunological sex-sorting protocol is required to minimize stress, although these mechanisms remain speculative and warrant further investigation.

In accordance with Thongkham et al. (2021) [14], imaging flow cytometry using Hoechst 33342 successfully discriminated X- and Y-chromosome-bearing sperm based on differences in DNA content, producing a characteristic double Gaussian distribution. In the present study, the sex-sorting process effectively shifted the natural 1:1 X:Y ratio observed in conventional semen. The supernatant fraction showed a marked enrichment of X-bearing sperm, reaching approximately 77–81%, whereas the eluted fraction was highly enriched with Y-bearing sperm, exceeding 83%. These findings indicate that the method can reliably bias sperm sex ratios in chilled boar semen, although some inter-boar variability remains. One limitation of this study is the relatively small number of boars (*n* = 3), which was constrained by animal availability. Although multiple ejaculates per boar were analyzed to enhance data robustness, further studies with a larger population are warranted to confirm the generalizability of these findings. However, further improvements are required to ensure consistent sperm quality, particularly in the Y-enriched fraction, before large-scale field application can be considered. In particular, protocol refinement and fertility trials are necessary to determine the suitability of this approach for commercial use.

## 5. Conclusions

The scFv antibody-based sex-sorting method using HL magnetic microbeads effectively discriminated between X- and Y-chromosome-bearing sperm in chilled porcine semen. The sex-sorting process resulted in a clear shift in sperm sex ratios, with the supernatant fraction enriched in X-sperm and the eluted fraction highly enriched in Y-sperm, confirming the efficiency of the separation technique. The preservation of motility, membrane integrity, and mitochondrial membrane potential in the X-enriched fraction indicates that this approach can maintain functional sperm quality compatible with practical application, whereas the reduced kinematic performance and physiological integrity observed in the Y-enriched fraction Importantly, the consistent outcomes obtained across individual Landrace boars demonstrate that the antibody, originally developed against Duroc-derived sperm antigens, retains effective binding performance across breeds, thereby highlighting the cross-breed applicability of the immunological sex-sorting technical. These findings indicate that HL magnetic bead-based immunological sex-sorting represents a promising and scalable approach for controlled sex selection in pigs, particularly for the production of X-enriched semen; however, further optimization and functional fertility validation are required to improve the post-sorting quality and practical utility of the Y-enriched fraction prior to large-scale application.

## Figures and Tables

**Table 1:** Sperm counting and % binding in the supernatant fraction and eluted fraction.

	Concentration of H4L4 Antibody (mg)			
	0	2	4	8
Before sexing	10.21	10.20	10.26	10.23
Supernatant fraction	10.03	4.98	4.97	5.13
Eluted fraction	0.17	5.19	5.20	5.00
Binding (%) *	1.66	50.83	50.73	48.89

* % Binding = (Eluted fraction sperm/total sperm) × 100.

**Table 2:** Proportions of X- and Y-bearing sperm in the supernatant and eluted fractions following separation with microbeads conjugated to three concentrations of H4L4 antibodies, as determined by imaging flow cytometry (mean ± SD).

Concentration of H4L4 scFv Antibody (mg/mL)	Supernatant Fraction		Eluted Fraction	
	X-Sperm	Y-Sperm	X-Sperm	Y-Sperm
0	51.2 ± 1.35 ^b^	48.8 ± 1.35 ^a^	49.2 ± 2.12 ^a^	50.8 ± 2.12 ^b^
2	78.4 ± 3.31 ^a^	21.6 ± 3.31 ^b^	22.6 ± 3.14 ^b^	77.4 ± 3.41 ^a^
4	79.3 ± 2.31 ^a^	20.7 ± 2.31 ^b^	21.9 ± 4.22 ^b^	78.1 ± 4.22 ^a^
8	78.1 ± 2.44 ^a^	21.9 ± 2.44 ^b^	22.2 ± 3.25 ^b^	77.8 ± 3.25 ^a^

Different superscript letters in the same column indicate significant differences (*p* < 0.05).

**Table 3:** The X/Y-sperm ratio in chilled sexed boar semen, evaluated by imaging flow cytometry.

Boar No.	CON		Supernatant Fraction		Eluted Fraction	
	X-Sperm	Y-Sperm	X-Sperm	Y-Sperm	X-Sperm	Y-Sperm
#1	50.1 ± 0.13 ^bA^	49.9 ± 0.13 ^bA^	79.8 ± 1.25 ^aA^	21.2 ± 1.25 ^cA^	16.5 ± 1.01 ^cA^	83.5 ± 1.01 ^aA^
#2	50.3 ± 0.24 ^bA^	49.7 ± 0.24 ^bA^	81.2 ± 2.45 ^aA^	18.8 ± 2.45 ^cA^	16.2 ± 1.25 ^cA^	83.2 ± 1.25 ^aA^
#3	50.4 ± 0.42 ^bA^	49.8 ± 0.42 ^bA^	77.4 ± 2.11 ^aA^	22.6 ± 2.11 ^cA^	27.4 ± 1.66 ^cB^	77.2 ± 1.66 ^aB^

Values are expressed as mean ± SD. Different lowercase superscript letters ^(a–c)^ within the same row indicate significant differences among fractions within the same boar. Different uppercase superscript letters ^(A–B)^ within the same column indicate significant differences among boars (*p* < 0.05).

**Table 4:** Assessment of sperm motility and kinematics in HL bead-sexed boar semen.

Factor		TM (%)	PM (%)	VCL [μm/s]	VSL [μm/s]	VAP [μm/s]	BCF [Hz]	HAC [rad]	WOB
Treatment	CON	78.22 ± 2.52	72.77 ± 2.03	90.92 ± 5.77	23.48 ± 2.74	31.28 ± 3.41	16.35 ± 1.54	0.34 ± 0.04	0.24 ± 0.03
	X-enriched	76.67 ± 4.13	70.14 ± 2.62	79.95 ± 4.30	21.16 ± 3.44	28.86 ± 2.95	16.78 ± 1.13	0.33 ± 0.04	0.33 ± 0.02
	Y-enriched	62.47 ± 3.20	50.71 ± 12.85	58.51 ± 4.02	10.90 ± 3.75	17.32 ± 3.96	10.33 ± 1.78	0.22 ± 0.02	0.28 ± 0.04
*p*-Value		<0.001	<0.001	<0.001	<0.001	<0.001	<0.001	<0.001	<0.001
Block	Boar 1	69.67 ± 2.72	56.31 ± 1.31	64.467 ± 2.81	16.04 ± 3.40	22.03 ± 3.29	13.11 ± 1.51	0.26 ± 0.02	0.22 ± 0.03
	Boar 2	73.01 ± 3.52	68.19 ± 3.56	76.15 ± 4.41	18.15 ± 2.84	25.49 ± 4.11	15.42 ± 1.21	0.30 ± 0.07	0.30 ± 0.02
	Boar 3	74.67 ± 3.60	69.12 ± 12.63	88.76 ± 6.86	21.35 ± 3.69	29.94 ± 2.93	14.93 ± 1.73	0.33 ± 0.01	0.33 ± 0.04
*p*-Value		0.061	0.083	0.068	0.065	0.162	0.097	0.088	0.067

**Table 5:** Flow cytometry results showing the membrane integrity of semen samples after sexing.

Factor		MI Living	MI Dead
Treatment	CON	83.07 ± 2.29	15.18 ± 2.09
	X-enriched	81.57 ± 2.90	15.61 ± 1.91
	Y-enriched	50.85 ± 3.82	44.90 ± 4.18
*p*-Value		<0.001	<0.001
Block	Boar 1	72.15 ± 2.82	25.15 ± 2.85
	Boar 2	65.11 ± 2.24	32.63 ± 2.44
	Boar 3	70.56 ± 2.90	27.30 ± 2.58
*p*-Value		0.077	0.055

MI—Membrane integrity.

**Table 6:** Mitochondrial membrane potential of sexed semen samples analyzed by flow cytometry.

Factor		MMP High	MMP Low
Treatment	CON	84.81 ± 3.88	15.12 ± 3.50
	X-enriched	79.38 ± 2.77	20.58 ± 2.41
	Y-enriched	56.11 ± 3.12	43.87 ± 2.40
*p*-Value		<0.001	<0.001
Block	Boar 1	69.68 ± 3.71	30.26 ± 2.13
	Boar 2	69.82 ± 2.56	30.13 ± 2.68
	Boar 3	73.08 ± 2.81	26.84 ± 2.93
*p*-Value		0.083	0.069

MMP—Mitochondrial membrane potential.

## Data Availability

The original contributions presented in this study are included in the article. Further inquiries can be directed to the corresponding author.

## References

[1] Vonderohe C.E., Brizgys L.A., Richert J.A., Radcliffe J.S. (2022). Swine Production: How Sustainable Is Sustainability?. Anim. Front..

[2] Kim S.W., Gormley A., Jang K.B., Duarte M.E. (2024). Current Status of Global Pig Production: An Overview and Research Trends. Anim. Biosci..

[3] Piao S., Jin X., Hu S., Lee J.-Y. (2024). The Impact of African Swine Fever on the Efficiency of China’s Pig Farming Industry. Sustainability.

[4] Mutua F., Dione M. (2021). The Context of Application of Biosecurity for Control of African Swine Fever in Smallholder Pig Systems: Current Gaps and Recommendations. Front. Vet. Sci..

[5] Woonwong Y., Do Tien D., Thanawongnuwech R. (2020). The Future of the Pig Industry After the Introduction of African Swine Fever into Asia. Anim. Front..

[6] Needham T., Lambrechts H., Hoffman L.C. (2017). Castration of Male Livestock and the Potential of Immunocastration to Improve Animal Welfare and Production Traits: Invited Review. S. Afr. J. Anim. Sci..

[7] Yodrug T., Hayakijkosol O., Wongtawan T. (2025). Two Decades of Sperm Sex-Sorting in Animals: A Comprehensive Systematic Review and Meta-Analysis of Techniques, Effectiveness, and Global Research Trends (2005–2025). Vet. World.

[8] Garner D.L. (2006). Flow Cytometric Sexing of Mammalian Sperm. Theriogenology.

[9] Maxwell W.M.C., Long C.R., Johnson L.A., Dobrinsky J.R., Welch G.R. (1998). Advances in Flow Cytometry for Sperm Sexing. Reprod. Fertil. Dev..

[10] Tiptiri-Kourpeti A., Asimakopoulos B., Nikolettos N. (2025). A Narrative Review on the Sperm Selection Methods in Assisted Reproductive Technology: Out with the New, the Old Is Better?. J. Clin. Med..

[11] Grossfeld R., Klinc P., Sieg B., Rath D. (2005). Production of Piglets with Sexed Semen Employing a Non-Surgical Insemination Technique. Theriogenology.

[12] Thongkham M., Hongsibsong S., Mekchay S., Sathanawongs A., Cao X.-M., Xu Z.-L., Sringarm K. (2025). Novel Single-Chain Fragment Variable Antibody Targeting Plasma Membrane Epitopes on Porcine Y-Chromosome-Bearing Sperm. Sci. Rep..

[13] Thongkham M., Hongsibsong S., Mekchay S., Sathanawongs A., Paitoon P., Saenjaiban A., Satsook A., Jantanasakulwong K., Rachtanapun P., Xu Z.-L. (2025). Revolutionizing Sexed Sorting Sperm Using ScFv Antibodies Combined with Microbeads for Porcine Sexed Semen. Int. J. Biol. Macromol..

[14] Thongkham M., Thaworn W., Pattanawong W., Teepatimakorn S., Mekchay S., Sringarm K. (2021). Spermatological Parameters of Immunologically Sexed Bull Semen Assessed by Imaging Flow Cytometry, and Dairy Farm Trial. Reprod. Biol..

[15] Thongkham M., Saenjaiban A., Jantanasakulwong K., Pattanawong W., Arjin C., Hongsibsong S., Rachtanapun P., Sringarm K. (2024). New Insights from Poly-Lactic Acid and Ionomer Films Coupled with Recombinant Antibodies for Processing Sexed-Sorting Bovine Sperm. Int. J. Biol. Macromol..

[16] Lacalle E., Fernández-Alegre E., Soriano-Úbeda C., Martínez-Martínez S., Domínguez J.C., González-Montaña J.R., Morrell J.M., Martínez-Pastor F. (2023). Single Layer Centrifugation (SLC) for Bacterial Removal with Porcicoll Positively Modifies Chromatin Structure in Boar Spermatozoa. Theriogenology.

[17] Paitoon P., Sartsook A., Thongkham M., Sathanawongs A., Lumsangkul C., Pattanawong W., Hongsibsong S., Sringarm K. (2024). Sperm Quality Variables of Sex-Sorted Bull Semen Produced by Magnetic-Activated Cell Sorting Coupled with Recombinant Antibodies Targeting Y-Chromosome-Bearing Sperm. Theriogenology.

[18] Sharpe J.C., Evans K.M. (2009). Advances in Flow Cytometry for Sperm Sexing. Theriogenology.

[19] Seidel G.E. (2003). Sexing Mammalian Sperm—Intertwining of Commerce, Technology, and Biology. Anim. Reprod. Sci..

[20] von Borell E., Baumgartner J., Giersing M., Jäggin N., Prunier A., Tuyttens F.A.M., Edwards S.A. (2009). Animal Welfare Implications of Surgical Castration and Its Alternatives in Pigs. Animal.

[21] Quelhas J., Pinto-Pinho P., Lopes G., Rocha A., Pinto-Leite R., Fardilha M., Colaço B. (2023). Sustainable Animal Production: Exploring the Benefits of Sperm Sexing Technologies in Addressing Critical Industry Challenges. Front. Vet. Sci..

[22] Johnson L.A., Rath D., Vazquez J.M., Maxwell W.M.C., Dobrinsky J.R. (2005). Preselection of Sex of Offspring in Swine for Production: Current Status of the Process and Its Application. Theriogenology.

[23] Bai L., Zhao Y., Zhou Y., Song Y., Xiao H., Zhao G., Wang Z., Li X. (2025). Advances in Immunological Sorting of X and Y Chromosome-Bearing Sperm: From Proteome to Sex-Specific Proteins. Front. Vet. Sci..

[24] Chot O., Thongkham M., Satsook A., Arjin C., Mekchay S., Hongsibsong S., Sringarm K. (2025). Production of Monoclonal Antibodies Targeting Plasma Membrane of Porcine Y-Chromosome-Bearing Sperm. Reprod. Biol..

[25] Chot O., Thongkham M., Satsook A., Paitoon P., Arjin C., Mekchay S., Sathanawongs A., Hongsibsong S., Sringarm K. (2026). Efficiency of Magnetic-Activated Cell Sorting Using Y-Specific Monoclonal Antibodies on the Quality of Porcine Sexed Semen. Theriogenology.

[26] Tanga B.M., Qamar A.Y., Raza S., Bang S., Fang X., Yoon K., Cho J. (2021). Semen Evaluation: Methodological Advancements in Sperm Quality-Specific Fertility Assessment—A Review. Anim. Biosci..

[27] Ribas-Maynou J., Barranco I., Salas-Huetos A. (2023). Sperm Quality and Fertility of Livestock Animals. Animals.

[28] Waberski D., Suarez S.S., Henning H. (2022). Assessment of Sperm Motility in Livestock: Perspectives Based on Sperm Swimming Conditions in Vivo. Anim. Reprod. Sci..

[29] Klein E.K., Swegen A., Gunn A.J., Stephen C.P., Aitken R.J., Gibb Z. (2022). The Future of Assessing Bull Fertility: Can the ’omics Fields Identify Usable Biomarkers?. Biol. Reprod..

[30] de Oliveira Leme L., Carvalho J.O., Mendes C.M., Assumpção M.E.O.D.A., Caetano A.R., Franco M.M., Dode M.A.N. (2024). Impact of Sperm Sex Sorting on Sperm Quality and in Vitro Embryo Production in Bovine. Anim. Reprod. Sci..

[31] Rath D., Moench-Tegeder G., Taylor U., Johnson L.A. (2009). Improved Quality of Sex-Sorted Sperm: A Prerequisite for Wider Commercial Application. Theriogenology.

[32] Leahy T., Gadella B.M. (2011). Sperm Surface Changes and Physiological Consequences Induced by Sperm Handling and Storage. Reproduction.

[33] Paitoon P., Sartsook A., Thongkham M., Chot O., Sathanawongs A., Pattanawong W., Sringarm K. (2024). Evaluation of Quality and Sex Ratio of Sperm in Post-Thaw Sexed Brahman Semen Using Magnetic-Activated Cell Sorting Conjugated with Y-ScFv Antibodies (M-Zlex). Vet. Integr. Sci..

